# Editorial Notes

**Published:** 1894-01

**Authors:** 


### Editorial Notes.

Dr. L. A. Felder, of Perry, Ga., has located in ourcity. We are glad to have such men as he to move to our city.

Dr. Frank H. Sims has resigned the professorship of diseases of the eye, ear, nose and throat in the Atlanta Polyclinic.

DISSOLUTION OF PARTNERSHIP.—The firm of Hummel & Parmele is dissolved and in future Dr. A. L. Hummel will conduct the business of advertising agent at 257 N. Fourth St., Philadelphia.

Dr. J. A. Weaver, of Buena Vista, Ga., was married to Miss Rene T. Dassinger on December, 26th, 1893. Dr. Weaver carried off the first honors in the Atlanta Medical College in class 1891.

Dr. W. C. Jarnigan and Miss Eskine Richmond, both of this city, were married on January 17th. Dr. Jarnigan is one of our leading physicians, and we wish them a very happy and prosperous life.

Dr. Luther B. Grandy, Editor of the *Atlanta Medical and Surgical Journal*, was married to Miss Hattie Smart, of this city, on December 14th, 1893. THE RECORD extends heartiest congratulations to the young couple.

TWO NEW MEDICAL JOURNALS.—"Mathews' Medical Quarterly," devoted to Diseases of the Rectum and Gastro-Intestinal Diseases, Rectal and Gastro-Intestinal Sugery, Joseph M. Mathews, M. D., Editor, Henry E. Tuly, M. D., Assistant Editor and Manager, Louisville, Ky., will be issued as soon as possible after January 1, 1894. Address, P. O. Box 434, Louisville, Ky.

GONE TO NEW YORK.—Dr. Robert L. Tye, of McDonough, Ga., left yesterday for New York, where he will spend several months in the hospitals of that city. From New York he will go to Europe and prepare for a specialty.

---

We are in receipt of the first issue of the Polyclinic Bulletin, published monthly by the Atlanta Polyclinic and edited by Dr. Arthur Childs. We extend a cordial welcome to our new neighbor and hope it will attain all the success its genial editor and publishers desire.

---

The Richmond Academy of Medicine holds semi-monthly meetings in the Y. M. C. A. building, Main and Sixth streets, every second and fourth Tuesday nights. All members of the profession visiting the city are always welcomed. For the year 1894, Dr. J. Spottwood Wellford was unanimously elected President, and Drs. John F. Woodward and Mark W. Peyser were re-elected Secretary and Assistant Secretary respectively.

---

THE ATLANTA SOCIETY OF MEDICINE.—At the last meeting in December the following officers were elected for the ensuing year:

President, Dr. J. W. Duncan; Vice-President, Dr. C. D. Hurt; Secretary, Dr. R. R. Kime; Treasurer, Dr. L. B. Grandy; Corresponding Secretary and Libarian, Dr. N. O. Harris;

---

MEDICAL ASSOCIATION OF GEORGIA.—The next meeting of this Association will be held in this city on April 18-19-20, with the following officers:

President, W. H. Elliott, Savannah; 1st Vice-Presihent, G. T. Miller, Americus; 2nd Vice-President, H. McHatton, Macon; Secretary, Dan H. Howell, Atlanta; Treasurer, E. C. Goodrich, Augusta. The medical profession of the State are invited to attend. The prospects are that we will have the largest meeting here in the history of the Association.

The local doctors extend a cordial invitation to all "regular" doctors of the State to visit us during the meeting.

"The Physician's Magazine," a Monthly Chronicle of the Advances of the Medical Sciences, edited by Robert C. Kenner, A. M., M, D., Louisville, Ky., and published by George F. Russell & Co., 808 First Street, Louisville, Ky., will be issued on January 1, 1894.

---

AMERICAN MEDICAL PUBLISHERS' ASSOCIATION.—The First Annual Meeting of this Association was held in the Grand Hotel, Cincinnati, on Monday, December 4th, 1893, and steps were taken in the direction of active, routine work. The by-laws and rules were revised and amended, while the name was modified in accordance with a demand from medical publishers of a general nature who desired to become members of the Association. The active co-operation of every medical publisher is earnestly solicited. Next meeting in Washington, D. C., September, 1894. Officers: President, Dr. Landon B. Edwards, Richmond, Va.; Vice-President, Dr. J. C. Culbertson, Cincinnati, Ohio.

